# To: Out-of-bed extubation: a feasible study

**DOI:** 10.5935/0103-507X.20150071

**Published:** 2015

**Authors:** Jacobo Bacariza Blanco, Antonio M. Esquinas

**Affiliations:** Intensive Care Unit, Hospital Garcia de Orta, EPE - Almada, Portugal.; Intensive Care Unit, Hospital Morales Meseguer - Murcia, Spain.

**To the Editor,**

Weaning from mechanical ventilation represents one of the major challenges and concerns in intensive care units worldwide. The withdrawal time represents at least 40% of the overall mechanical ventilation period. Furthermore, in 30% of clinical cases some incidents will force the clinician to stop the attempt. Fortunately, there have been substantial improvements in mechanical ventilation weaning since release of the weaning and discontinuation ventilation guidelines in 2001,^(1)^ standardizing the clinical practice of weaning protocols. Sedation control, adjusting doses to the minimum amount needed, daily spontaneous breathing trials after satisfying the respiratory assessment criteria and chest physical therapy (inspiratory muscle strength training) in the early stages of the illness, to avoid ventilator-induced diaphragm dysfunction (VIDD), are the cornerstones of current weaning protocols intended to avoid secondary wean failure (SWF). However, there remain many questions on this topic that merit further investigation.

Dexheimer Neto et al. make progress in addressing these as yet unanswered questions.^(2)^ The goal was to assess the advantages of extubating a patient after mobilization in an unusual position (seated in an arm chair) compared with the regular practice of extubation in the supine position. They concluded that there were no differences in the results for the two groups, (seated versus supine position).^(1)^

With respect to the three main tools intended to prevent SWF mentioned above, the most poorly understood at present is chest physical therapy. Although controversy still exists because of limited data and a lack of multicenter trials on chest physical therapy,^(3)^ it seems pathophysiologycally^(4)^ that this therapy, in association with uncontrolled ventilator modalities, would significantly reduce the incidences of muscle atrophy, structural injury and respiratory muscle fiber remodeling, thereby preventing VIDD and failure to wean. However, the problem extends beyond the specific type of therapy, to when and how to use it. Chest physical therapy protocols are needed to solve this problem. These protocols should be tailored to address the main named causes of VIDD; however, we cannot forget cost effectiveness, respiratory secretion control and general motor muscle training. These are additional major concerns related to and causes of SWF that can interfere with weaning. Pharmacologic therapies such as expectorants, mucolytics, mucokinetics and mucoregulators and non-pharmacologic therapies such as humidification (active or passive), percussion and cough assist (manually or mechanically), forced expiratory technique, and intrapulmonary percussive ventilation, although controversial, have been shown to improve the airway. As a result, they have some beneficial effects on pulmonary function, gas exchange, oxygenation and length of stay.^(5)^ To summarize, chest physical therapy protocols should be developed to improve respiratory outcomes before extubation and create the best conditions for preventing failure to wean. Instead of focusing on how or where we extubate a patient we have focused on the approach we take to preparing the patient for extubation. However, further randomized clinical trials and research studies are needed to investigate these issues. Additionally, Dexheimer Neto et al.^(2)^ improved our understanding of the proper conditions for extubation with their excellent paper.

*Jacobo Bacariza Blanco* *Intensive Care Unit, Hospital Garcia de Orta, EPE - Almada,
Portugal.**Antonio M. Esquinas* *Intensive Care Unit, Hospital Morales Meseguer - Murcia,
Spain.*

## References

[1] MacIntyre NR, Cook DJ, Ely EW, Epstein SK, Fink JB, Heffner JE, Hess D, Hubmayer RD, Scheinhorn DJ, American College of Chest Physicians, American Association for Respiratory Care, American College of Critical Care Medicine (2001). Evidence-based guidelines for weaning and discontinuing
ventilatory support: a collective task force facilitated by the American
College of Chest Physicians; the American Association for Respiratory Care;
and the American College of Critical Care Medicine. Chest.

[2] Dexheimer FL, Vesz PS, Cremonese RV, Leães CG, Raupp AC, Rodrigues Cdos S (2014). Out-of-bed extubation: a feasible study. Rev Bras Ter Intensiva.

[3] Daniel Martin A, Smith BK, Gabrielli A (2013). Mechanical ventilation, diaphragm weakness and weaning: a
rehabilitation perspective. Respir Physiol Neurobiol.

[4] Sigala I, Vassilakopoulos T, Esquinas A (2008). Respiratory muscle fatigue (requiring rest to recover) is the
cause of weaning failure. Yearbook respiratory care clinics and applied technologies.

[5] Andrews J, Sathe NA, Krishnaswami S, McPheeters ML (2013). Nonpharmacologic airway clearance techniques in hospitalized
patients: a systematic review. Respir Care.

