# Supplementary material for: To: Out-of-bed extubation: a feasible study
Source: Rev Bras Ter Intensiva. 2015 Oct-Dec;27(4):419–21. doi: 10.5935/0103-507X.20150071 (PMC4738832; doi:10.5935/0103-507X.20150071)
Supplement: Related texts

# Reply: AUTHORS’ RESPONSE

**Authors:** Felippe Leopoldo Dexheimer Neto

**Affiliations:** Intensive Care Unit, Hospital Moinhos de Vento - Porto Alegre (RS), Brazil; Intensive Care Unit, Hospital Mãe de Deus - Porto Alegre (RS), Brazil.

We thank you for your letter and compliments. Improvements in weaning are crucial to avoiding both unnecessary prolongation of mechanical ventilation and premature extubation because both are related to an increased risk of complications in critically ill patients.^(1)^

As you highlighted, weaning from mechanical ventilation has significantly improved in the last 15 years due to the implementation of weaning protocols.^(1)^ Less sedation, daily trials of spontaneous breathing, and physical therapy in the earliest days of a critical illness have changed our current practice; now, the intensive care unit (ICU) team faces new questions.^(1-3)^ When we designed our study, we focused on one specific issue, which was related to how we should schedule our routine care.^(4)^

Recently, a systematic review was performed that evaluated the efficacy of interventions targeting physical functioning (PF) among ICU survivors.^(5)^ As you noted, none of the available trials studying medications or devices showed better outcomes for long-term PF. The conclusion of this systematic review was that the only effective intervention for improving long-term PF is exercise/physical therapy.^(5)^

However, most of the reports included individualized physical therapy programs that varied in intensity and frequency, from subject to subject.^(5)^ There are many physiological theories attempting to explain the potentially synergistic effects of these therapies on muscle strength; however, questions regarding the best type and timing (early therapy seems better) of therapy are unanswered.^(5,6)^ Unfortunately, another important barrier to rehabilitation may be the lack of physical therapists.^(5,6)^

In conclusion, the implementation of protocols for preventing ICU-acquired weakness, which combine daily sedation interruption, spontaneous breathing and early physical therapy, are associated with a shorter duration of mechanical ventilation.^(1,3)^ Supporting and expanding upon your statement, our study was not only about where to extubate a patient but also emphasized that early exercise and mobilization during daily periods of sedation interruption are the best options for preparing the patient for optimal recovery. However, as you indicated, further research is necessary.

*Felippe Leopoldo Dexheimer Neto* *Intensive Care Unit, Hospital Moinhos de Vento - Porto Alegre (RS),
Brazil; Intensive Care Unit, Hospital Mãe de Deus - Porto Alegre
(RS), Brazil.*

## References

[1] Peñuelas Ó, Thille AW, Esteban A (2015). Discontinuation of ventilatory support: new solutions to old
dilemmas. Curr Opin Crit Care.

[2] Hodgson CL, Stiller K, Needham DM, Tipping CJ, Harrold M, Baldwin CE (2014). Expert consensus and recommendations on safety criteria for
active mobilization of mechanically ventilated critically ill
adults. Crit Care.

[3] Balas MC, Vasilevskis EE, Olsen KM, Schmid KK, Shostrom V, Cohen MZ (2014). Effectiveness and safety of the awakening and breathing
coordination, delirium monitoring/management, and early
exercise/mobility bundle. Crit Care Med.

[4] Dexheimer FL, Vesz PS, Cremonese RV, Leães CG, Raupp AC, Rodrigues CS (2014). Out-of-bed extubation: a feasibility study. Rev Bras Ter Intensiva.

[5] Calvo-Ayala E, Khan BA, Farber MO, Ely EW, Boustani MA (2013). Interventions to improve the physical function of ICU
survivors: a systematic review. Chest.

[6] Walsh CJ, Batt J, Herridge MS, Santos CC (2014). Muscle wasting and early mobilization in acute respiratory
distress syndrome. Clin Chest Med.
